# Flexible boosting of accelerated failure time models

**DOI:** 10.1186/1471-2105-9-269

**Published:** 2008-06-06

**Authors:** Matthias Schmid, Torsten Hothorn

**Affiliations:** 1 Institut für Medizininformatik, Biometrie und Epidemiologie, Friedrich-Alexander-Universität Erlangen-Nürnberg, Waldstraße 6, D-91054 Erlangen, Germany; 2 Institut für Statistik, Ludwig-Maximilians-Universität München, Ludwigstraße 33, D-80539 München, Germany

## Abstract

**Background:**

When boosting algorithms are used for building survival models from high-dimensional data, it is common to fit a Cox proportional hazards model or to use least squares techniques for fitting semiparametric accelerated failure time models. There are cases, however, where fitting a fully parametric accelerated failure time model is a good alternative to these methods, especially when the proportional hazards assumption is not justified. Boosting algorithms for the estimation of parametric accelerated failure time models have not been developed so far, since these models require the estimation of a model-specific scale parameter which traditional boosting algorithms are not able to deal with.

**Results:**

We introduce a new boosting algorithm for censored time-to-event data which is suitable for fitting parametric accelerated failure time models. Estimation of the predictor function is carried out simultaneously with the estimation of the scale parameter, so that the negative log likelihood of the survival distribution can be used as a loss function for the boosting algorithm. The estimation of the scale parameter does not affect the favorable properties of boosting with respect to variable selection.

**Conclusion:**

The analysis of a high-dimensional set of microarray data demonstrates that the new algorithm is able to outperform boosting with the Cox partial likelihood when the proportional hazards assumption is questionable. In low-dimensional settings, i.e., when classical likelihood estimation of a parametric accelerated failure time model is possible, simulations show that the new boosting algorithm closely approximates the estimates obtained from the maximum likelihood method.

## Background

Predicting the expected time to event from a high-dimensional set of predictor variables has become increasingly important in the last years. A particularly interesting problem in this context is the analysis of studies relating patients' genotypes, for example measured via gene expression levels, to a clinical outcome such as "disease free survival" or "time to progression". Survival models of this type share the common problems that are typical for the analysis of gene expression data: Sample sizes are small while the number of potential predictors (i.e., gene expression levels) is extremely large. As a consequence, standard estimation techniques can not be applied any more.

For these reasons, a variety of new methods for obtaining survival predictions from high-dimensional data have been suggested in the literature. Most of these methods are focused on the Cox proportional hazards model [1], while some other methods have been developed for fitting semiparametric accelerated failure time (AFT) models [2] in high-dimensional settings. Tibshirani [3], Gui and Li [4], Park and Hastie [5], and Zou [6] introduced Lasso-like algorithms for minimizing *L*_1 _penalized versions of the Cox partial likelihood. Due to the structure of the *L*_1 _penalty, these methods have the advantage that variable selection is carried out simultaneously with parameter estimation. Li and Luan [7] introduced a technique for maximizing the *L*_2 _penalized Cox partial likelihood, which (in contrast to methods using the *L*_1 _penalty) does not carry out variable selection but includes all predictors. On the other hand, several authors developed methods for fitting semiparametric AFT models in high-dimensional data settings: While Huang and Harrington [8] and Wang et al. [9] considered modifications of the Buckley-James method [10], Huang et al. [11] and Datta et al. [12] developed algorithms based on a censoring-adjusted penalized least squares loss function. In addition to penalized estimation techniques, there are various strategies for reducing the dimensionality of microarray data *before *building an unpenalized survival model, see Schumacher et al. [13], Bovelstad et al. [14], and van Wieringen et al. [15] for overviews of this topic.

In this paper the focus is on gradient boosting [16,17], which is an alternative method for fitting survival models in high-dimensional data settings. Similar to the Lasso, boosting has a built-in variable selection mechanism which is carried out simultaneously with the estimation of the prediction function. Although being connected to the Lasso (see Efron et al. [18]), boosting algorithms are not primarily designed for the maximization of penalized (partial) likelihood functions but can rather be interpreted as a method for minimizing convex loss functions via gradient descent techniques. In each step of a boosting algorithm, a so-called *base-learner *(e.g., a linear regression model) is fitted to the negative gradient of a pre-specified loss function. The current estimate of the prediction function is then updated with the newly obtained estimate of the gradient. As the base-learner can be modified such that only one covariate is used for estimating the gradient in each step (leading to the so-called *component-wise base-learner*), variable selection is carried out at each iteration.

Originally introduced for classification problems by Freund and Schapire [19,20], boosting has developed into a computationally efficient technique for fitting many types of regression models in high-dimensional data settings. In principle, the boosting loss function can be any negative log likelihood function of some exponential family. Various authors have shown that the method tends to be promising with respect to both variable selection and prediction accuracy [16,17,21,22].

Concerning the prediction of survival outcomes from gene expression data, the development of boosting algorithms has so far focused on the same model families as *L*_1 _and *L*_2 _penalized estimation techniques, namely the Cox proportional hazards model and the semiparametric AFT model. Ridgeway [23], Li and Luan [24], and Binder and Schumacher [25] used boosting algorithms with the negative partial log likelihood loss function while Hothorn et al. [26] introduced a boosting algorithm for fitting semiparametric AFT models. Similar to Huang et al. [11], Hothorn et al. [26] used censoring weights for adjusting the least squares objective function.

While the proportional hazards model is the most frequently used survival model in biostatistics and while fitting semiparametric AFT models is a robust method for survival prediction when there is unknown heterogeneity in the data, application of both types of analyses is still inadequate in a number of situations. Instead, it might be advisable to fit a fully *parametric *AFT model with an explicitly specified distribution of the survival outcome, such as the Weibull or the log-logistic distribution [2].

As an example we consider a high-dimensional set of microarray data originally analyzed in a classification context by Barrier et al. [27]. The authors collected a sample of 50 patients operated on for a stage II colon adenocarcinoma. 25 patients developed a metachronuous metastasis, whereas the other 25 patients remained disease free for at least 60 months. For each patient the expressions of 22,283 genes were obtained. Barrier et al. [27] selected a sample consisting of the 30 most differentially expressed genes between the disease and the disease-free group. By applying diagonal linear discriminant analysis based on the expressions of the 30 genes, the authors achieved a high prediction accuracy (76.3%) for the two patient groups. In the following we will address the equally important problem of modeling the *time *to development of metachronuous metastasis.

**Figure 1:**
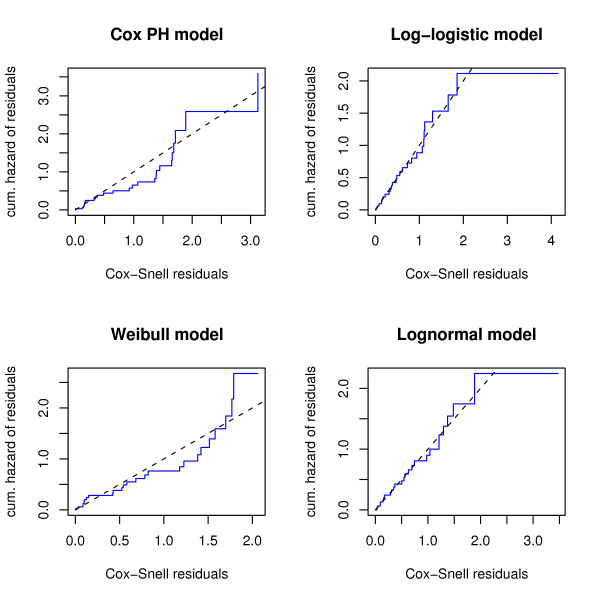
**Cox-Snell residuals obtained from fitting various survival models to the Barrier data**. The upper left panel shows the Cox-Snell residuals of a semiparametric Cox model vs. the Nelson-Aalen estimate of their cumulative hazard function. Estimates were obtained from fitting a Cox proportional hazards model to the Barrier data via maximization of the partial log likelihood. The 14 most differentially expressed genes between the disease and the disease-free group were used as predictor variables. The other panels show the Cox-Snell residuals (together with their cumulative hazard function) obtained from fitting various parametric AFT models to the same data via maximum likelihood estimation. Obviously, the lines corresponding to the Cox-Snell residuals of the log-logistic and lognormal models are closest to the line through the origin, indicating that these models fit the data best. By contrast, the Cox model and the Weibull model (which both assume proportional hazards) do not seem to fit the data well, indicating that the proportional hazards assumption is violated.

**Figure 2:**
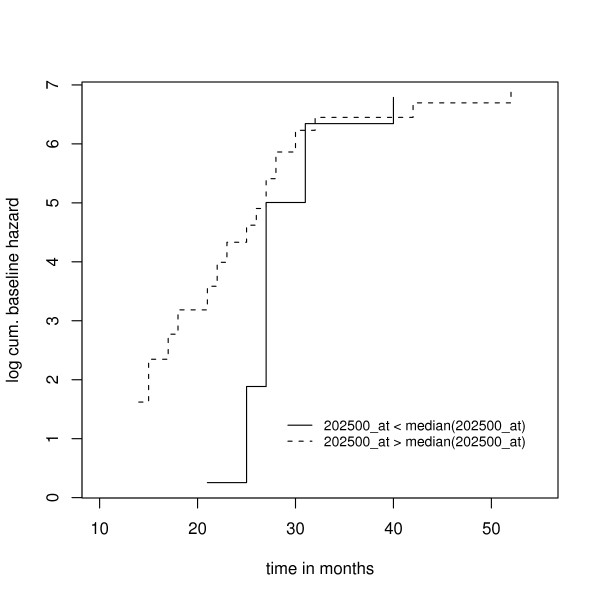
**Estimated log cumulative hazard functions obtained from fitting a stratified Cox model to the Barrier data**. Estimates were obtained via maximization of the stratified partial log likelihood. The strata were generated by splitting the expression values of the most overexpressed gene in the disease group (202500_at) at their median. The remaining 13 of the 14 most differentially expressed genes were used as predictor variables in the stratified Cox model.

Figs. 1 and 2 show the results of a preliminary survival analysis of the Barrier stage II colon cancer data: Here only the 14 most differentially expressed genes between the disease and the disease-free group (i.e., the genes with p-values less than or equal to 0.005) were used as predictor variables. The Cox-Snell residuals shown in Fig. 1 suggest that a parametric AFT model with either a log-logistic or a lognormal distribution fits the data best. The Cox proportional hazards model does not seem to be appropriate, which is further confirmed by the results shown in Fig. 2: Here the parameters of a stratified Cox model were estimated, where the strata were defined by splitting the values of the most overexpressed gene in the disease group at their median. The two baseline hazard functions shown in Fig. 2 cross, so the proportional hazards assumption seems to be violated.

Although the preliminary analysis is by no means sufficient for building a survival model from the Barrier data, we have nevertheless gained valuable insight into the distribution of the survival times. How should we incorporate this "prior knowledge" into a boosting algorithm? Fig. 2 suggests that using the Cox partial likelihood as a loss function for boosting is at least questionable, since the Cox model is known to be sensitive with respect to violations of the proportional hazards assumption (see Schemper [28]). On the other hand, Fig. 1 suggests that the survival times of the Barrier data either follow a log-logistic distribution or a lognormal distribution, so fitting a semiparametric AFT model without any distributional assumption might be inefficient (given that we only have 50 observations with 50% of them being censored). In order to account for these issues, we focus on the development of a boosting algorithm for parametric AFT models. An algorithm of this type has not yet been developed in the literature, since AFT models include a scale parameter for modeling the variance of the error distribution in the regression equation. With maximum likelihood estimation of AFT models, this scale parameter is estimated simultaneously with the regression parameters. Boosting algorithms, however, have so far not been able to deal with scale parameters but only with the prediction function, i.e., with the regression parameters. This is the reason why it has not been possible yet to use the negative log likelihood function of an AFT model as a loss function for boosting. In fact, boosting Cox models and semiparametric AFT models was only possible because these methods do not include a scale parameter.

In the following we will introduce a new boosting algorithm that allows for simultaneous estimation of both the prediction function and the scale parameter in parametric AFT models. This algorithm uses the negative log likelihood of the AFT model as a loss function and works in a stepwise fashion. After starting with some offset values of the regression and scale parameters, a component-wise base-learning procedure is applied to the negative gradient in each iteration, and an update of the prediction function is obtained. Afterwards, the scale parameter is re-estimated in each iteration by plugging the current estimate of the prediction function into the loss function and by minimizing the loss function over the scale parameter. As a base-learning procedure we use the classical linear least squares approach, i.e., the final model can be interpreted as a survival fit depending on a linear predictor.

The characteristics of the new algorithm are first investigated by means of a simulation study with artificial data. It is shown that variable selection is carried out efficiently, and that the algorithm has a high predictive power if compared to the "null models" with no covariates at all. Also, the regression estimates of boosting in low-dimensional settings turn out to be almost identical to the corresponding maximum likelihood estimates. To show the practical applicability of the new algorithm, we carry out a detailed study on the above introduced data set by Barrier et al. [27]. Evaluating the performance measures of this study suggests that boosting with the negative log likelihood of a parametric AFT model is able to outperform boosting with the Cox partial log likelihood, at least in case of the Barrier data.

## Methods

### Estimation problem

Consider a set of realizations of i.i.d. random variables (*T*_1_, *C*_1_, *X*_1_),..., (*T*_*n*_, *C*_*n*_, *X*_*n*_), where *T*_1_,..., *T*_*n *_are one-dimensional survival times, *C*_1_,..., *C*_*n *_are one-dimensional censoring times, and *X*_1_,..., *X*_*n *_are *p*-dimensional vectors of covariates. It is assumed that some of the survival times *T*_1_,..., *T*_*n *_are right-censored, so that only the random variables ${\tilde{Y}}_{i}$ := min{*T*_*i*_, *C*_*i*_}, *i *= 1,..., *n*, are observable. This implies that the available data consist of realizations of ${\tilde{Y}}_{i}$ and of the set of censoring indicators *δ*_*i *_∈ {0, 1}, where *δ*_*i *_= 0 if observation *i *is censored and *δ*_*i *_= 1 if the complete survival time *T*_*i *_has been observed. It is further assumed that the *C*_*i*_, *i *= 1,...,*n*, are independent of (*T*_*i*_, *X*_*i*_), *i *= 1,...,*n*. This corresponds to the classical case of "random censoring". The objective is to fit the accelerated failure time model

(1) log(*T*) = *f*(*X*) + *σW*,

where (*T*, *X*) follows the same distribution as each of the (*T*_*i*_, *X*_*i*_), *i *= 1,...,*n*, and where *W *is a random noise variable independent of *X*. The function *f *is an unknown prediction function of log(*T*), and *σ *is an unknown scale parameter that controls the amount of noise added to the prediction function *f*. Typically, *f *is a linear or additive function of the covariates *X*.

The distributional assumption on the noise variable *W *determines the distributional form of the survival time *T*. If this distribution is fully specified, we refer to Model (1) as a *parametric *AFT model. For example, if *W *follows a standard extreme value distribution, *T *(given *X*) follows a Weibull distribution with parameters λ:= exp(- *f*/*σ*) and *α*:= 1/*σ *(cf. Klein and Moeschberger [29]). Other popular examples of parametric AFT models are the log-logistic distribution for *T*|*X *(with *W *following a standard logistic distribution) and the lognormal distribution for *T*|*X *(with *W *following a standard normal distribution). It is important to note that the latter two models explicitly allow for non-proportional hazards. The Weibull distribution is a parametric example of the well-known Cox proportional hazards model (which does not necessarily assume a regression equation such as (1) but is instead based on a semiparametric model for the hazard function of *T*|*X*).

Classically, the estimation of *f *and *σ *in a parametric AFT model is performed by maximizing the log likelihood function

(2) $$\sum_{i=1}^{n}\delta_{i}\left[-\log\sigma +\log f_{W}\left(\frac{Y_{i}-f(X_{i})}{\sigma }\right)\right]+\left(1-\delta_{i}\right)\left[\log S_{W}\left(\frac{Y_{i}-f(X_{i})}{\sigma }\right)\right],$$

where *Y*_*i *_:= log(min{*T*_*i*_, *C*_*i*_}) is the logarithm of ${\tilde{Y}}_{i}$. The functions *f*_*W *_and *S*_*W *_denote the probability density and survival functions of the noise variable *W*, respectively (cf. Klein and Moeschberger [29], Chapter 12).

*Semiparametric *AFT models leave the parameter *σ *and the distribution of the noise variable *W *in Model (1) unspecified. Instead of using maximum likelihood techniques, *f *is estimated via minimization of a censoring-adjusted least squares criterion [2,10,30].

### FGD boosting with component-wise linear least squares and scale parameter estimation

In the following we will use boosting techniques for obtaining precise estimates of Model (1) when the number of covariates is large. We start by considering a boosting algorithm known as "component-wise linear functional gradient descent (FGD)" [16,17,21,31]. The objective of FGD is to obtain the real-valued prediction function

(3) f* := argmin_*f*(·)_E [*ρ*(*Y*, *f*(*X*))],

where the one-dimensional function *ρ *is a convex loss function that is assumed to be differentiable with respect to *f*. Estimation of f* is performed by minimizing the empirical risk $\sum_{i=1}^{n}\rho (Y_{i},f(X_{i}))$ with respect to *f*. Component-wise FGD works as follows:

1. Initialize the *n*-dimensional vector ${\hat{f}}^{\left[0\right]}$ with an offset value, e.g., ${\hat{f}}^{\left[0\right]}\equiv 0$. Set *m *= 0.

2. Increase *m *by 1. Compute the negative gradient $-\frac{\partial }{\partial f}\rho (Y,f)$ and evaluate at ${\hat{f}}^{\left[m-1\right]}(X_{i})$, *i *= 1,...,*n*. This yields the negative gradient vector

$$U^{\left[m-1\right]}={\left(U_{i}^{\left[m-1\right]}\right)}_{i=1,...,n}:={\left(-\frac{\partial }{\partial f}\rho {\left(Y,f\right)|}_{Y=Y_{i},f={\hat{f}}^{\left[m-1\right]}(X_{i})}\right)}_{i=1,...,n}.$$

3. Fit the negative gradient *U*^[*m*-1] ^to each of the *p *components of *X *(i.e., to each covariate) separately by *p *times using a simple linear regression estimator. This yields *p *estimates of the negative gradient vector *U*^[*m*-1]^.

4. Select the component of *X *which fits *U*^[*m*-1] ^best according to a pre-specified goodness-of-fit criterion. Set ${\hat{U}}^{\left[m-1\right]}$ equal to the fitted values from the corresponding best model fitted in Step 3.

5. Update ${\hat{f}}^{\left[m\right]}={\hat{f}}^{\left[m-1\right]}+\nu {\hat{U}}^{\left[m-1\right]}$, where 0 <*ν *≤ 1 is a real-valued step length factor.

6. Iterate Steps 2–5 until *m *= *m*_stop _for some stopping iteration *m*_stop_.

The above algorithm can be interpreted as a negative gradient descent algorithm in function space. In each step, an estimate of the true negative gradient of the loss function is added to the current estimate of f*. Thus, a structural (regression) relationship between *Y *and the covariate vector *X *is established (for details on the characteristics of FGD we refer to Bühlmann and Hothorn [22]). Moreover, FGD carries out variable selection in each iteration, as only one component of *X *is selected in Step 4. This property makes the algorithm applicable even if *p *> *n*. Due to the additive structure in Step 5, the final boosting estimate at iteration *m*_stop _can be interpreted as a linear model fit but will typically depend on only a subset of the *p *components of *X*.

As we want to use FGD for the estimation of parametric AFT models, we set *ρ *equal to the negative log likelihood function specified in (2). However, in this case, the standard FGD algorithm presented above can not be applied, as (2) includes an additional scale parameter *σ *that has to be estimated simultaneously with *f*. We therefore extend the classical FGD algorithm in the following way:

1. Initialize the *n*-dimensional vector ${\hat{f}}^{\left[0\right]}$ with an offset value, e.g., ${\hat{f}}^{\left[0\right]}\equiv 0$. *In addition, initialize the one-dimensional scale parameter *${\hat{\sigma }}^{\left[0\right]}$*with an offset value, e.g*., ${\hat{\sigma }}^{\left[0\right]}=1$. Set *m *= 0.

2. Increase *m *by 1. Compute the negative gradient $-\frac{\partial }{\partial f}\rho (Y,f,\sigma )$ and evaluate at ${\hat{f}}^{\left[m-1\right]}(X_{i})$, *i *= 1,...,*n*, *and at *${\hat{\sigma }}^{\left[m-1\right]}$. This yields the negative gradient vector

$$U^{\left[m-1\right]}={\left(U_{i}^{\left[m-1\right]}\right)}_{i=1,...,n}:={\left(-\frac{\partial }{\partial f}\rho {\left(Y,f,\sigma \right)|}_{Y=Y_{i},f={\hat{f}}^{\left[m-1\right]}(X_{i}),\sigma ={\hat{\sigma }}^{\left[m-1\right]}}\right)}_{i=1,...,n}.$$

3. Fit the negative gradient *U*^[*m*-1] ^to each of the *p *components of *X *(i.e., to each covariate) separately by *p *times using a simple linear regression estimator. This yields *p *estimates of the negative gradient vector *U*^[*m*-1]^.

4. Select the component of *X *which fits *U*^[*m*-1] ^best according to a pre-specified goodness-of-fit criterion. Set ${\hat{U}}^{\left[m-1\right]}$ equal to the fitted values from the corresponding best model fitted in Step 3.

5. Update ${\hat{f}}^{\left[m\right]}={\hat{f}}^{\left[m-1\right]}+\nu {\hat{U}}^{\left[m-1\right]}$, where 0 <*ν *≤ 1 is a real-valued step length factor. *Plug *${\hat{f}}^{\left[m\right]}$*into the empirical risk function *$\sum_{i=1}^{n}\rho (Y_{i},f(X_{i}),\sigma )$*and minimize the empirical risk over σ*. *This yields the scale parameter estimate *${\hat{\sigma }}^{\left[m\right]}$.

6. Iterate Steps 2–5 until *m *= *m*_stop _for some stopping iteration *m*_stop_.

It is easily seen that the modified FGD algorithm estimates f* and *σ *in a stepwise fashion: In Steps 3 and 4, f* is estimated for a given value of *σ*, while in Step 5, *σ *is estimated given the current estimate of f*. It is also clear from Step 4 that the built-in variable selection mechanism of FGD is not affected by the additional estimation of the scale parameter *σ*. The value of the stopping iteration *m*_stop _is the main tuning parameter of FGD. In the following we will throughout use five-fold cross-validation for determining the value of *m*_stop_, i.e., *m*_stop _is the iteration with lowest predictive risk. The choice of the step-length factor *ν *has been shown to be of minor importance for the predictive performance of a boosting algorithm. The only requirement is that the value of *ν *is "small", such that a stagewise adaption of the true prediction function f* is possible (see Bühlmann and Hothorn [22]). We therefore set *ν *= 0.1. As a goodness-of-fit criterion in Step 4 we use the *R*^2 ^measure of explained variation which is known from linear regression.

### Measures for the predictive power of boosting techniques

After having built a survival model from a set of microarray data, it is essential to evaluate the predictive performance of the model. To this purpose, various measures of predictive accuracy have been proposed in the literature [32-37]. Since none of these measures has been adopted as a standard so far, we use the following strategy for measuring the performance of a survival prediction rule:

#### a) Log-Likelihood

If the objective is to compare prediction rules obtained from the *same *model family (such as the family of Weibull distributions), we use the predictive log likelihood or partial log likelihood as a measure of predictive accuracy. The predictive log likelihood or partial log likelihood is defined as follows: Suppose that the parameter vector *θ *of a survival model has been estimated from a training sample, and that a test sample (*T*_*k*_, *C*_*k*_, *X*_*k*_), *k *= 1,...,*n*_test_, is used for evaluating the predictive accuracy of the model. Denote the log likelihood or partial log likelihood function of the survival model by *l*_*θ*_(*T*, *C*, *X*). The predictive log likelihood is then given by

(4) $$l_{\text{pred}}(\hat{\theta }):=\sum_{k=1}^{n_{\text{test}}}l_{\hat{\theta }}(T_{k},C_{k},X_{k}),$$

where the parameter estimate $\hat{\theta }$ has been plugged into the log likelihood or partial log likelihood of the test sample. In case of a parametric AFT model we have *θ *= (*f*, *σ*), whereas in case of a Cox model, *θ *is the prediction function *f *used for modeling the hazard rate of *T*|*X*. In a boosting context it is particularly advisable to use (4) as a measure of predictive accuracy, since the negative log likelihood or partial log likelihood is used as a loss function for the corresponding boosting algorithms. As a consequence, the loss function is measured on the same scale as the predictive accuracy (4).

#### b) Brier-Score

If the objective is to compare prediction rules obtained from different model families or estimation techniques, it is no longer possible to use the predictive log likelihood as a measure of predictive accuracy. This is because the likelihood and partial likelihood functions of different model families are usually measured on different scales, so they can not be compared directly. Moreover, many nonparametric and semiparametric estimation techniques do not involve likelihood functions at all, implying that a predictive likelihood value can not be computed.

In case of the Barrier data we will compare the performance of various parametric and semiparametric estimation techniques by using the Brier score [34,37] as a measure of predictive accuracy. The Brier score is defined as the time-dependent squared distance between the predicted survival probability and the true state of an observation. Since the Brier score is not based on any specific model assumptions, the predictive accuracy of various model families and estimation techniques can be measured on the same scale. Also, possible misspecifications of a survival model are taken into account [37]. In this paper we use the methodology of Gerds and Schumacher [38] who combined the evaluation of the Brier score with the 0.632+ estimator developed by [39]. This methodology has been used previously for predicting survival outcomes from microarray data sets [25].

We first draw *B *bootstrap samples of size *n *from the data, where the bootstrapped observations constitute the training data sets and the out-of-bootstrap observations constitute the test data sets. Define Γ_*i*_(*t*) := I(*T*_*i *_> *t*), *i *= 1,...,*n*, for each time point *t *> 0. Further denote by $\hat{S}(t,X_{i})$, *i *= 1,...,*n*, the survival function of observation *i *at time point *t *estimated from the complete data set. We compute the apparent error rate

(5) $$\text{err}(t):=\frac{1}{n}\sum_{i=1}^{n}{\left(\Gamma_{i}(t)-\hat{S}(t,X_{i})\right)}^{2}W_{i}(t,\hat{G}),$$

where

(6) $$W_{i}(t,\hat{G}):=\frac{I({\tilde{Y}}_{i}\leq t)\delta_{i}}{\hat{G}({\tilde{Y}}_{i}-)}+\frac{I({\tilde{Y}}_{i}>t)}{\hat{G}(t)}$$

are weights that are introduced to account for censoring [37]. The expression $\hat{G}$ denotes the Kaplan-Meier estimate of the distribution of the censoring times *C*_*i*_, *i *= 1,...,*n*. For each bootstrap sample and for each time point *t *we further compute the out-of-bag error rates

(7) $${\text{Err}}_{b}(t):=\frac{1}{n_{b}}\sum_{i\epsilon {ℬ}_{b}}^{n}{\left(\Gamma_{i}(t)-{\hat{S}}_{b}(t,X_{i})\right)}^{2}W_{i}(t,\hat{G}),\;b=1,...,B,$$

where *n*_*b *_is the cardinality of the set ${ℬ}_{b}$ of out-of-bootstrap observations corresponding to bootstrap sample *b*, *b *= 1,..., *B*. The expression ${\hat{S}}_{b}(t,X_{i})$ is the estimated survival function of the out-of-bootstrap observation *i *at time point *t *obtained from the bootstrap training sample *b*. Denote the cross-validated error rate, i.e., the mean or median of the out-of-bag error rates Err_*b*_(*t*), *b *= 1,...,*B*, by ${\text{Err}}_{ℬ}(t)$.

Both (5) and (7) are estimators of the true prediction error E[Γ(*t*) - *S*(*t, X*)]^2^, where *S*(*t, X*) is the true survival function of *T*|*X *at time point *t*. Since (7) is an upward-biased estimator and (5) is a downward-biased estimator of the true prediction error (see Gerds and Schumacher [38]), we use a linear combination of err(*t*) and ${\text{Err}}_{ℬ}(t)$ as the overall measure of accuracy at time point *t*:

(8) $$\text{Err}(t):=[1-\omega (t)]\text{err}(t)+\omega (t){\text{Err}}_{ℬ}(t).$$

Defining *ω*(*t*) := 0.632/(1 - 0.368*R*(*t*)) with $R(t):=\frac{{\text{Err}}_{ℬ}(t)-\text{err}(t)}{\text{NoInferr}(t)-\text{err}(t)}$ and

(9) $$\text{NoInferr}(t):=\frac{1}{n^{2}}\sum_{i=1}^{n}\sum_{j=1}^{n}{\left(\Gamma_{i}(t)-\hat{S}(t,X_{j})\right)}^{2}W_{i}(t,\hat{G})$$

yields the 0.632+ estimator of the Brier score at time point *t*. For a detailed derivation of (9) we refer to Gerds and Schumacher [38] and Efron and Tibshirani [39]. In case of the Barrier data, we evaluate (9) at each time point *t *> 0 up to a survival time of five years (i.e., *t*_max _= 60 months). This yields a prediction error curve depending on *t*, where 0 <*t *≤ *t*_max_.

## Results and Discussion

In this section we investigate the properties of the new boosting algorithm for parametric AFT models. We first conduct a simulation study with artificial data. Afterwards, we investigate the ability of boosting to predict survival outcomes from gene expression data. This is done by conducting a benchmark study on the Barrier stage II colon cancer data. All computations were carried out with the R system for statistical computing (version 2.6.2, [40]) using a modification of the glmboost() function in package mboost (version 1.0–1, [41]).

### Simulation study with artificial data

We carried out a simulation study on linear AFT models with five covariates, i.e., we considered the model

(10) log(*T*) = *β*_1_*X*_1 _+ ... + *β*_1_*X*_5 _+ *σW*,

where *X*_1_,...,*X*_5 _are jointly normally distributed covariates with parameters E(*X*_*k*_) = 0, var(*X*_*k*_) = 1, and cov(*X*_*k*_, *X*_*l*_) = 0.5, *k*, *l *= 1,... 5, *k *≠ *l*. The data values of the noise variable *W *were either drawn from a standard extreme value distribution (corresponding to the Weibull model), from a standard logistic distribution (corresponding to the log-logistic model), or from a standard normal distribution (corresponding to the lognormal model). For each of the three distributions the scale parameter *σ *was chosen such that

(11) $$\frac{\mathrm{var}(\beta_{1}X_{1}+\ldots +\beta_{1}X_{5})}{\mathrm{var}(\beta_{1}X_{1}+\ldots +\beta_{1}X_{5})+\mathrm{var}(\sigma W)}=0.8,$$

i.e., the linear predictor *f*(*X*) accounted for 80% of the variance of log(*T*). The parameter vector *β*:= (*β*_1_,...,*β*_5_)^⊤ ^was set equal to *β *= (0.5, 0.25, —0.25, —0.5, 0.5)^⊤^.

In a first step, we compared the estimates of *β *obtained from the new boosting algorithm with the corresponding estimates obtained from standard maximum likelihood estimation. For each of the three distributions of *W*, we generated 50 i.i.d. data sets (of size *n *= 100 each) from Model (10). Censoring was introduced to the data by simulating 50 additional i.i.d. data sets (of size 100 each) from Model (10). The values of the dependent variables in these additional data sets constituted the censoring times of the corresponding first 50 data sets. This implied that (a) the censoring times followed the same distribution as the survival times but were independent from the latter, and that (b) about 50% of the observations in each data set were censored on average. The new boosting algorithm with the corresponding negative log likelihood loss function was applied to each data set. Note that the final boosting estimate can be interpreted as a linear model fit (depending on a parameter estimate of *β*), since we used component-wise *linear *base-learners. We additionally used standard maximum likelihood techniques for estimating the parameters of Model (10) from the 50 data sets.

**Figure 3:**
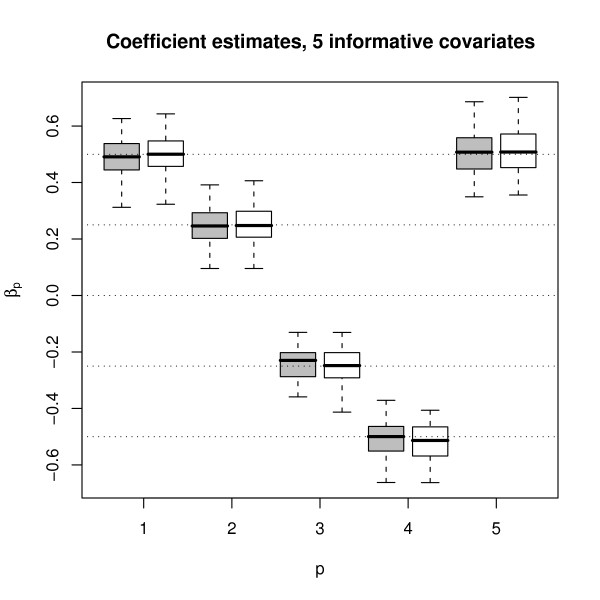
**Boxplots of the Weibull parameter estimates when 5 informative covariates are present**. Boxplots of the estimates of *β *= (0.5, 0.25, -0.25, -0.5, 0.5)^⊤^, as obtained from the 50 Weibull-distributed samples following Model (10). Grey boxplots correspond to boosting estimates, white boxplots correspond to maximum likelihood estimates. Similar results were obtained for the log-logistic and lognormal models.

**Figure 4:**
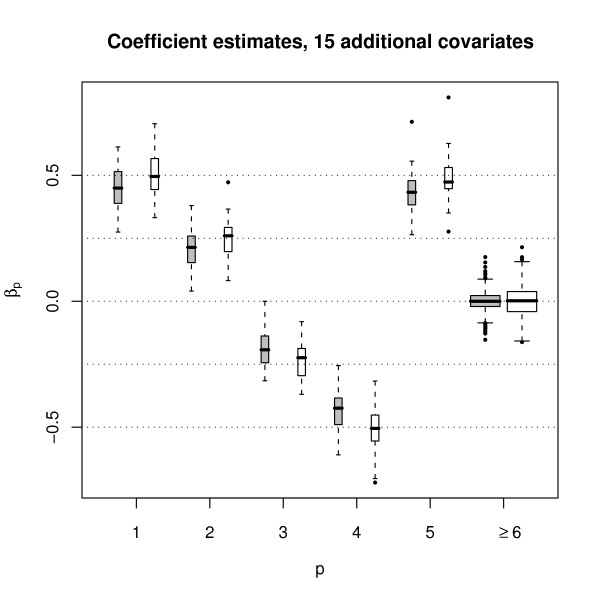
**Boxplots of the Weibull parameter estimates when 5 informative and 15 additional non-informative covariates are present**. Boxplots of the estimates of *β*_1_,...,*β*_20_, as obtained from the 50 Weibull-distributed samples following Model (10). Grey boxplots correspond to boosting estimates, white boxplots correspond to maximum likelihood estimates. Similar results were obtained for the log-logistic and lognormal models.

The boxplots of the parameter estimates of the Weibull model are shown in Fig. 3. Obviously, the parameter estimates obtained from boosting are very similar to the parameter estimates obtained from maximum likelihood estimation. Similar results were obtained for the log-logistic and lognormal models. This can also be seen from the Spearman correlations between the parameter estimates shown in Table 1. We next compared the ability of boosting and of standard maximum likelihood techniques to select a subset of informative covariates out of a larger set of covariates. To this purpose, we extended the set of variables used for the previous simulation study by an additional set of 15 independent standard normally distributed covariates with parameters *β*_6 _= ... = *β*_20 _= 0. Fig. 4 shows the resulting parameter estimates obtained from boosting and from maximum likelihood estimation. Obviously, all estimates of the "non-informative" parameters *β*_6_,...,*β*_20 _are close to the true value of 0. In 33.7% of all 15 · 50 cases, the boosting parameter estimates of *β*_6_,...,*β*_20 _are exactly 0, which means that non-informative covariates have not been selected. The corresponding percentage rates were 32.1% and 21.5% for the log-logistic and the lognormal model, respectively. In addition, the boosting parameter estimates corresponding to *β*_6_,...,*β*_20 _have a smaller variance than the corresponding maximum likelihood estimates. For these reasons, boosting seems to be preferable to likelihood estimation when it comes to variable selection. It can also be seen from Fig. 4 that the boosting estimates of the non-zero parameters *β*_1_,...,*β*_5 _are (slightly) downward-biased. This shrinking effect reflects the well-known bias-variance trade-off which is common to boosting techniques.

**Table 1:** Spearman correlations between the parameter estimates of Model (10). Spearman correlations between the boosting estimates and the maximum likelihood estimates, as obtained from the 50 samples following Model (10).

	${\hat{\beta }}_{1}$	${\hat{\beta }}_{2}$	${\hat{\beta }}_{3}$	${\hat{\beta }}_{4}$	${\hat{\beta }}_{5}$
Weibull	0.9745	0.9710	0.9607	0.9568	0.9741
Log-logistic	0.9615	0.9700	0.9309	0.9506	0.9698
Lognormal	0.9980	0.9991	0.9986	0.9979	0.9983

**Figure 5:**
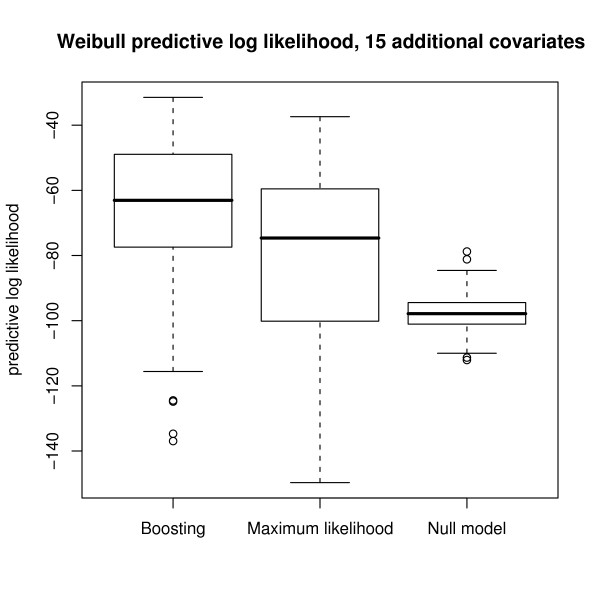
**Boxplots of the predictive Weibull log likelihood estimates**. Boxplots of the predictive Weibull log likelihood estimates, as obtained from the 50 Weibull-distributed test samples following Model (10). The predictive log likelihood values of the null model were obtained via maximum likelihood estimation with no covariates and an intercept only. Similar results were obtained for the log-logistic and lognormal models.

We finally investigated the *predictive *performance of the new boosting algorithm and of maximum likelihood estimation techniques. To this purpose, we used the model estimates obtained from the 50 data sets for predicting the likelihood of 50 additionally generated i.i.d. test samples of sample size *n*_test _= 100 each (which also followed Model (10)). The corresponding predictive log likelihood values, which are shown in Fig. 5, suggest that the predictive performance of boosting is better than the predictive performance of maximum likelihood estimation. A Wilcoxon paired-sample test for the predictive log likelihood values of boosting and maximum likelihood estimation resulted in a p-value of less than 0.001. For the log-logistic and lognormal models, the respective p-values were also smaller than 0.001.

### Benchmark experiment on stage II colon cancer data

In order to show the practical applicability of the new boosting algorithm for parametric AFT models, we conducted a benchmark study on the Barrier stage II colon cancer data [27]. Again we used (1) the negative Weibull log likelihood, (2) the negative log-logistic log likelihood, and (3) the negative lognormal log likelihood as loss functions for boosting. To compare boosting for parametric AFT models with other estimation techniques, we additionally fitted survival models by using (4) *L*_2_Boosting for semiparametric AFT models [26], (5) *L*_1 _penalized estimation for semiparametric AFT models [11] (using the lars() function in R package lars [42]), (6) gradient boosting with the negative Cox partial log likelihood loss [23], (7) *L*_1 _penalized Cox partial likelihood estimation (using the coxpath() function in R package glmpath [43]), and (8) nonparametric estimation of the survival function via the Kaplan-Meier estimator. The latter estimator corresponds to the non-informative "null model" with no covariates at all.

We applied the eight estimation techniques to 50 bootstrap training samples generated from the Barrier data. Prediction errors were obtained by using the 0.632+ methodology, as described in the Methods section. For computational reasons we reduced the predictor space for the *L*_1 _penalized methods (5) and (7), i.e., we used the 10,218 most differentially expressed genes between the disease and the disease-free group instead of all 22,283 genes. The subset of 10,218 genes corresponds to those genes whose differences between the disease and the disease-free group are significant at a level of *α *= 0.2. This reduction of the predictor space did not have any negative effect on the predictive performance of the two *L*_1 _penalized techniques (see later). The tuning parameters of all eight methods were determined by using five-fold cross validation.

The survival functions of the eight methods, which are needed for computing the Brier score, were estimated as follows: For the "parametric" boosting methods (1), (2), and (3), we estimated the survival functions by plugging the estimated prediction function $\hat{f}$ into the Weibull, log-logistic, and lognormal survival functions, respectively. In case of *L*_2_Boosting and *L*_1 _penalized estimation for semiparametric AFT models (methods (4) and (5)) we made use of the following relationship:

(12) *S*(*t*, *X*) = P(*T *> *t*|*X*) = P(*f*(*X*) + *ε *> log*(t*)|*X*) = P(*ε *> log(*t*) - *f*(*X*)|*X*),

where *ε *:= *σW*. We then estimated the probability on the right-hand side of (12) by applying the Kaplan-Meier estimator to the residuals ${\hat{\varepsilon }}_{i}$ obtained from the bootstrap training samples and by evaluating the Kaplan-Meier survival functions at "time points" $\text{log}(t)-\hat{f}(X_{i})$. In case of boosting with the Cox partial log likelihood loss and *L*_1 _penalized Cox regression (methods (6) and (7)) we used the estimated survival function

(13) $$\hat{S}(t,X)=\exp\left[-{\hat{\Lambda }}_{0}(t)\exp(\hat{f})\right],$$

where ${\hat{\Lambda }}_{0}(t)$ is the Breslow estimator of the cumulative baseline hazard of the survival times.

**Figure 6:**
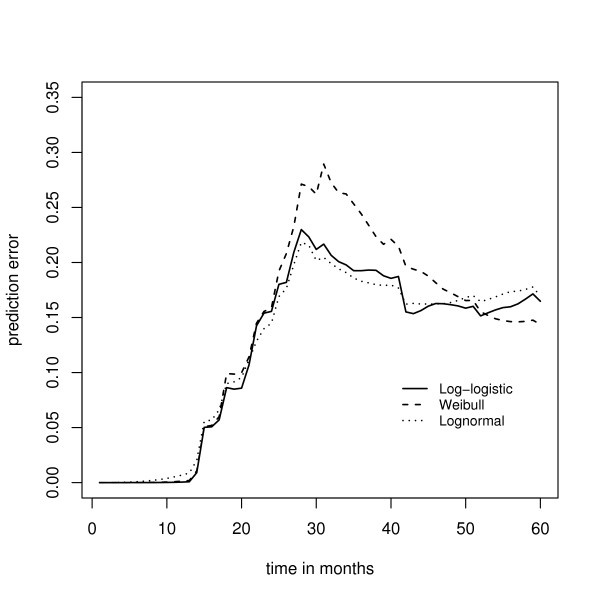
**Analysis of the Barrier stage II colon cancer data – prediction error curves for various parametric AFT models**. Prediction error curves obtained from boosting with the negative log-logistic log likelihood, boosting with the negative Weibull log likelihood, and boosting with the negative lognormal log likelihood.

**Figure 7:**
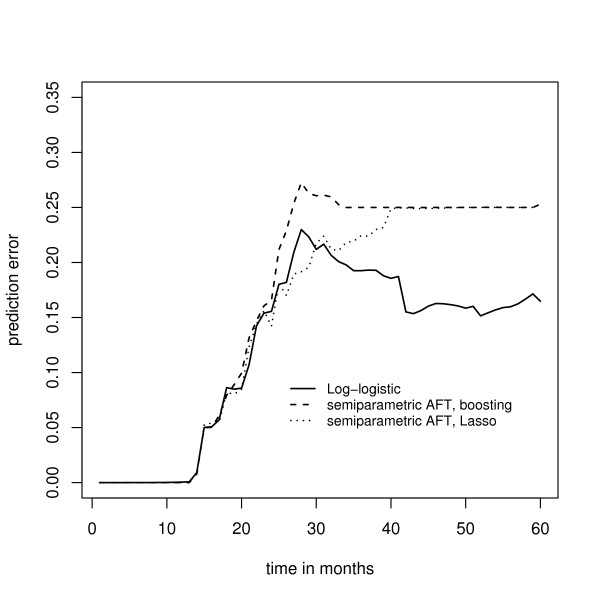
**Analysis of the Barrier stage II colon cancer data – prediction error curves for parametric and semiparametric AFT models**. Prediction error curves obtained from boosting with the negative log-logistic log likelihood, *L*_2_Boosting for semiparametric AFT models, and *L*_1 _penalized estimation for semiparametric AFT models (Lasso).

**Figure 8:**
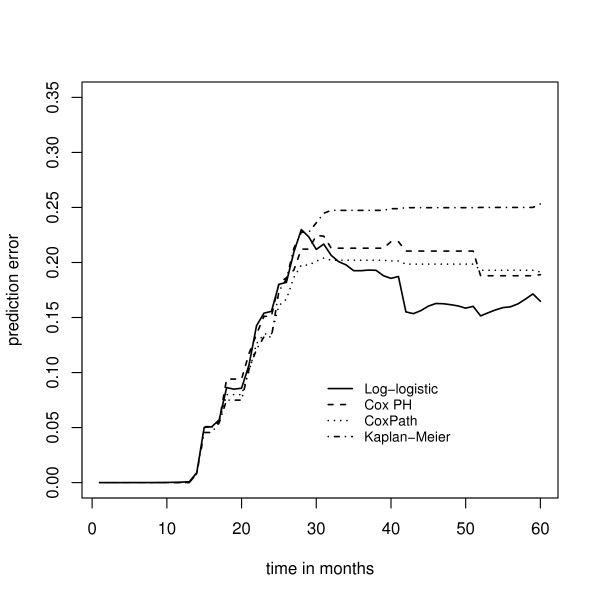
**Analysis of the Barrier stage II colon cancer data – prediction error curves for various survival models**. Prediction error curves obtained from boosting with the negative log-logistic log likelihood, boosting with the negative Cox partial log likelihood, *L*_1 _penalized estimation of a Cox proportional hazards model (CoxPath), and nonparametric estimation via the Kaplan-Meier estimator.

Fig. 6 shows the median prediction error curves corresponding to the parametric AFT boosting techniques (1), (2), and (3). Obviously, the prediction error curves cross, so the predictive performance of the models depends on the time point *t *under consideration. However, for most values of *t*, boosting with the negative log-logistic or negative lognormal log likelihood loss yields the smallest prediction error. In Fig. 7 the median prediction error curve corresponding to the log-logistic model is compared to the median prediction error curves obtained from the two techniques for semiparametric AFT models (4) and (5). Here the parametric AFT model seems to have the best predictive performance, indicating that the efficiency loss due to the semiparametric estimation of the AFT model is relatively large. In Fig. 8 the median prediction error curve of the log-logistic model is compared to the median prediction error curves obtained from the two partial log likelihood techniques (6) and (7), as well as to the median prediction error curve obtained from the "null model" (8). Again we see that boosting with the negative log-logistic log likelihood has the best predictive performance.

**Figure 9:**
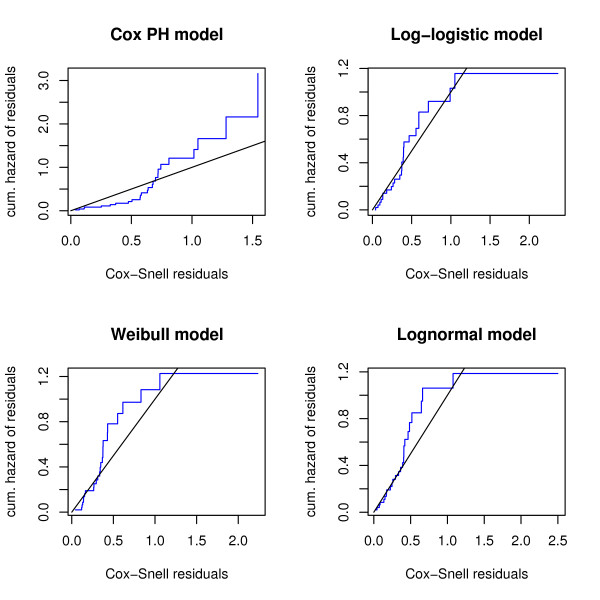
**Analysis of the Barrier stage II colon cancer data – Cox-Snell residuals for various boosting methods**. The upper left panel shows the Cox-Snell residuals of a semiparametric Cox model vs. the Nelson-Aalen estimate of their cumulative hazard function. Estimates were obtained from boosting with the negative Cox partial log likelihood. The other panels show the Cox-Snell residuals (together with their cumulative hazard function) obtained from fitting various parametric AFT models to the same data via boosting with the corresponding negative log likelihood loss. Similar to Fig. 1, we see that the line corresponding to the Cox-Snell residuals of the log-logistic model is close to the line through the origin. The Cox model does not seem to fit the data well, indicating that the proportional hazards assumption is violated.

We finally computed the Cox-Snell residuals from the boosting estimates based on the complete data set. The residuals, which are shown in Fig. 9, confirm the results obtained from the preliminary analysis of the Barrier data presented in the Background section: Similar to Fig. 1, we see that the log-logistic model fits the data well, while the Cox proportional hazards model seems to be inadequate here.

## Conclusion

By introducing a boosting algorithm for parametric AFT models we have extended the methodology for modeling survival times in high-dimensional data settings. Boosting algorithms for survival data have previously been developed for the Cox proportional hazards model [23,24] and for semiparametric AFT models [26]. An extension of these algorithms to parametric AFT models has not been possible so far, since parametric AFT models depend on a scale parameter which has to be estimated simultaneously with the prediction function. To overcome this problem we have developed a flexible boosting algorithm which is able to deal with loss functions depending on *both *the prediction function and a scale parameter. As a consequence, the negative log likelihood function of an AFT model can be used as a loss function for the component-wise functional gradient descent boosting algorithm.

The simulation study on various parametric AFT models has shown that the favorable properties of boosting with respect to variable selection are left untouched by the additional estimation of the scale parameter. Moreover, when sample sizes are small (such that a semiparametric estimation of AFT models becomes inefficient) or when the proportional hazards assumption of the survival times is violated, boosting techniques for parametric AFT models seem to lead to an increase in prediction accuracy. This is suggested by the results obtained from the benchmark study on Barrier's stage II colon cancer data. We finally point out that we have so far focused exclusively on boosting with the component-wise *linear *base-learning procedure. This procedure fits a set of simple linear regression models to the negative gradient in each boosting iteration. We have used component-wise linear least squares mainly because of their computational efficiency. In fact, component-wise linear least squares base-learners are highly efficient even when the number of predictors is extremely large (such as in case of Barrier's stage II colon cancer data, where *p *> 22,000). In principle, however, the new boosting algorithm can also be extended to additive models with smooth components. This can, for example, be done by using component-wise smoothing splines (see Bühlmann and Yu [17]) or P-splines as base-learners. Moreover, the boosting algorithm developed in this paper is not exclusively designed for fitting parametric AFT models but could also be used in combination with other likelihood-based loss functions depending on a scale parameter. The properties of these extensions still have to be investigated and constitute an issue for future research.

## Authors' contributions

MS developed the algorithm, conducted the benchmark experiments, and wrote the initial version of the manuscript. TH is the primary author of the mboost package, contributed to the design of the benchmark study and revised the manuscript.
